# Occurrence of Malignancies Other than Breast and Ovarian Cancer in Female Carriers of a *BRCA1/2* Germline Pathogenic Variant

**DOI:** 10.3390/cancers17101687

**Published:** 2025-05-17

**Authors:** Annechien Stuursma, Bert van der Vegt, Lieke P. V. Berger, Maaike B. C. ten Hoor, Jan C. Oosterwijk, Marian J. E. Mourits, Geertruida H. de Bock

**Affiliations:** 1 Department of Obstetrics & Gynecology, University Medical Center Groningen, University of Groningen, 9713 GZ Groningen, The Netherlands; 2 Department of Epidemiology, University Medical Center Groningen, University of Groningen, 9713 GZ Groningen, The Netherlands; g.h.de.bock@umcg.nl; 3 Department of Clinical Genetics, University Medical Center Groningen, University of Groningen, 9713 GZ Groningen, The Netherlands; 4 Department of Pathology & Medical Biology, University Medical Center Groningen, University of Groningen, 9713 GZ Groningen, The Netherlands; b.van.der.vegt@umcg.nl; 5 Department of Surgery, University Medical Center Groningen, University of Groningen, 9713 GZ Groningen, The Netherlands

**Keywords:** *BRCA1/2*, cancer, gastrointestinal, head and neck, female genitals

## Abstract

Introduction: Previous studies have suggested an additional increased risk for types of malignancies other than breast and ovarian cancer for female *BRCA1/2* GPV carriers, but risk estimates vary widely. The aim of this study was to investigate if female *BRCA1/2* GPV carriers have an increased risk of malignancies other than breast and tubal/ovarian cancer at an early age. Methods: Prospectively collected data from female *BRCA1/2* GPV carriers in our hospital-based data/biobank were linked to the PALGA Dutch Pathology Database. Incidences of malignancies occurring before 60 years of age were compared to crude rates/100.000 person-years in the Netherlands, stratified by age and calendar time. Standardized incidence ratios (SIRs) were calculated with 95% confidence intervals (95%CIs). Results: In 1347 women, 82 malignancies other than breast and tubal/ovarian cancer were detected in patients under 60 years of age in 37,068 person-years. An increased risk of cancer in general (SIR:2.25, 95%CI:1.78–2.80, *p* < 0.001), head and neck cancer (SIR:3.17, 95%CI:1.03–7.39, *p* < 0.05), gastrointestinal cancer (SIR:1.96, 95%CI:1.14–3.13, *p* < 0.05), and female genital cancer (SIR:2.48, 95%CI:1.61–3.65, *p* < 0.001) was found. Conclusions: If confirmed in larger, prospective studies that include the role of bias and previous cancer treatment, awareness of the possible increased risks of head and neck, gastrointestinal, and female genital cancer may be used to tailor clinical guidelines for female *BRCA1/2* GPV carriers.

## Simple Summary

Women with a *BRCA1/2* germline pathogenic variant (GPV) are at an increased risk of developing breast and ovarian cancer. Previous studies have suggested that they may also have an increased risk of developing other types of cancer at an early age. The aim of this study is to investigate this risk of developing other types of cancer before age 60. We linked data from our hospital-based data/biobank to data from the Dutch Nationwide Pathology Databank (PALGA). We included 1347 women with a *BRCA1/2* GPV who had 82 diagnosed cancers (other than breast and ovarian cancer) in our study. We found an increased risk of cancer in general, as well as an increased risk of head and neck cancer, gastrointestinal cancer, and female genital cancer (ovarian cancer excluded). However, larger studies that can account for other risk factors for cancer are necessary to confirm these results

## 1. Introduction

The *BRCA1* and *BRCA2* genes [*BRCA1/2*] are tumor suppressor genes that code for proteins involved in double-strand DNA damage repair [1]. Women with a *BRCA1/2* germline pathogenic variation [GPV] have an increased risk of developing breast and ovarian cancer that is diagnosed at an early age. The average cumulative risk of developing breast cancer by the age of 80 years is 72% [95%CI = 65–79] for *BRCA1* carriers and 69% [95%CI = 61–77%] for *BRCA2* GPV carriers, compared to a lifetime risk of 12.9% in the general population [2,3]. Breast cancer incidence increases rapidly in early adulthood until ages 30–40 for *BRCA1* carriers and ages 40–50 years for *BRCA2* GPV carriers. For tubal/ovarian cancer, the average cumulative risk by the age of 80 is 44% [95%CI = 36–53] for *BRCA1* and 17% [95%CI = 11–25] for *BRCA2* GPV carriers, compared to a lifetime risk of 1.2% in the general population [2,4].

Ovarian cancer mortality is reduced by up to 96% if a risk-reducing salpingo-oophorectomy [RRSO] is performed before ovarian incidence rises [age 35–40 years for *BRCA1* carriers and age 40–45 years for *BRCA2* GPV carriers] [5,6]. To reduce breast cancer-specific mortality, annual screening from the age of 25, including a physical examination and magnetic resonance imaging [MRI], in addition to [bi]annual mammography from the age of 40, is an effective option offered to GPV carriers [7,8]. Another option is risk-reducing mastectomy [RRM], which reduces breast cancer risk by 90–95%, although compared to annual screening, this has not yet translated into a survival benefit [9]. Moreover, treatment modalities have improved breast cancer-specific survival for *BRCA1/2*-related breast cancer over the past several years, with the most recent addition to treatment being the use of poly adenosine diphosphate [ADP]-ribose polymerase [PARP] inhibitors [10].

Given improved prevention and survival, knowledge regarding possible increased risks for other types of cancer at an early age in *BRCA1/2* GPV carriers is becoming increasingly relevant. Family-based studies that have included first-degree relatives with unknown mutation statuses may have underestimated the risks for proven *BRCA1/2* GPV carriers [11,12,13,14,15]. Many prospective studies that have solely included proven *BRCA1/2* GPV carriers have focused on investigating the risk of developing specific types of tumors, such as uterine or pancreatic cancer [16,17,18,19,20,21,22,23,24]. Risk estimates in the aforementioned studies have been inconsistent in size and direction. However, Li et al. [25] recently published separate risk estimates for various types of malignancies other than breast and ovarian cancer in males and females, GPV carriers and noncarriers, and untested relatives. For female *BRCA1* GPV carriers, a significantly increased risk was found for pancreatic and stomach cancer. For female *BRCA2* GPV carriers, an increased risk of bladder, lung, pancreatic, and stomach cancer was reported. Risks were particularly increased before the age of 65 years.

The aim of the current study is to determine whether Dutch women proven to be *BRCA1/2* GPV carriers have an increased risk of malignancies other than breast and ovarian cancer at an early age. We chose to investigate this risk before the age of 60 years and compared it to the general population. The current study will add to the body of evidence by providing relative risks specific to Dutch women proven to be *BRCA1/2* GPV carriers. Unbiased results will help to define optimal care for *BRCA1/2* GPV carriers.

## 2. Materials and Methods

### 2.1. OncoLifeS

For the purpose of this study, data from a hospital-based biobank at the University Medical Center of Groningen [UMCG] called OncoLifeS was used [26]. Since 1994, after written informed consent has been obtained, all women with a hereditary increased risk of breast and ovarian cancer have been prospectively included in this data/biobank, mainly women with a *BRCA1/2* GPV. In OncoLifeS, routine clinical data are linked to questionnaires and biological specimens. OncoLifeS has been approved by the medical ethics committee of the UMCG and was registered in the Dutch Trial Register under the following number: NL7839. The participation rate of this group of women who were asked to participate in OncoLifeS is about 95%. On the OncoLifeS consent form, permission is requested to link the responses to other data sources [26]. Data from OncoLifeS were linked to PALGA in January 2022, and at the same time, a link with the municipal registry was established to evaluate if the women were still alive, and if not, what their date of death was.

### 2.2. PALGA

PALGA is the Dutch nationwide network and registry of histopathology and cytopathology [27]. Data in this database consist of excerpts of malignancies diagnosed in the Netherlands, starting from the year 1971. Since 1 January 1989, PALGA has had national coverage. Excerpts can be requested from the research board of PALGA, after informed consent from study participants has been guaranteed. The available data consist of tumor type, grade, stage, depth, diameter, and differentiation grade, metastatic regions, lymph node involvement, immunohistochemistry data, and surgical margins, if applicable. For this study, excerpts were requested for all diagnosed malignancies in the study population [28].

### 2.3. Study Population

Women in the current study were selected from OncoLifeS if they had a proven *BRCA1* and/or *BRCA2* GPV and were aged 18 years or older at the time of data linkage. Data retrieved from OncoLifeS included mutation type, date of birth, date of diagnosis of breast cancer and/or ovarian cancer and date of death.

### 2.4. Outcomes

The primary outcome was defined as the overall incidence of malignancies other than breast and ovarian cancer in female *BRCA1/2* GPV carriers before age 60 [29,30,31]. We chose an arbitrary age limit of 60 years for the inclusion of tumors because one of the main characteristics of a hereditary predisposition to malignancies is a younger age at onset. The expected cancer incidence was available from the Netherlands Cancer Registry [NCR]. Female sex, age, calendar year, and country-specific incidence rates [crude/100.000 person-years] stratified by age and calendar time were obtained for the calendar years 1989–2016 [January 2023].

### 2.5. Categorization of Malignancies

Malignancies retrieved from PALGA reports were categorized based on the International Classification of Diseases for Oncology [ICD-O] [28]. Fallopian tube cancer [considered to be the likely origin of high-grade serous ovarian cancer] and peritoneal cancer were considered ovarian cancer and therefore were not analyzed as ‘other malignancies’ in this study. Basal cell carcinomas were not analyzed in the total group of skin cancers because they are not included in the data regarding the incidence of skin cancers derived from the Netherlands Cancer Registry. All other types of skin cancers were included in the primary analysis.

### 2.6. Statistical Analysis

Observation started from the first day of PALGA coverage [1 January 1989], or from the patient’s date of birth, whichever came last. Observation ended on the date of their 60th birthday, date of death, date of linkage [January 2022], or date of diagnosis of a tumor other than breast and/or ovarian cancer, whichever came first. Breast and ovarian cancers were not counted as tumors in this analysis, i.e., observation did not end on the date of breast cancer or ovarian cancer diagnosis.

Expected cancer incidence was estimated based on the calculated person-time at risk, stratified by female sex, age, and calendar time. Standardized incidence rates [SIRs] were calculated by dividing observed cancers by expected cancers and 95% confidence intervals [CIs], and 2-sided *p*-values were estimated by assuming a Poisson distribution for the observed number of cancers. *p*-values < 0.05 were considered statistically significant. Statistics were performed using SPSS-software, version 26, and STATA software, version 17.0.

## 3. Results

The characteristics of the 1347 women included in this study are described in Table 1. A total of 37,068 person-years at risk were available: 19,799 years for women with a *BRCA1* GPV and 17,269 years for women with a *BRCA2* GPV. The median age at the time of linkage with PALGA was 53.9 years. Women with a *BRCA1* GPV were 60.8 years old, and women with a *BRCA2* GPV were 46.6 years old. A total of 79 of the 1347 included women had a history of ovarian cancer [5.8%], and 520 women had a history of breast cancer [38.6%] at the time of linkage [January 2022].

A total of 82 malignancies other than breast and ovarian cancer were found in participants under the age of 60 years [6.9%], with a median of 6.3 years after breast cancer diagnosis [interquartile range [IQR]: 1.4 before to 16 years after breast cancer diagnosis, n = 47] and/or a median of 9.6 years after ovarian cancer diagnosis [IQR: 1.2 before to 13.9 after ovarian cancer diagnosis, n = 5]. The median age at which cancer, other than breast and ovarian cancer, was first observed before age 60 was 46.9 years.

Ten tumors developed after the women had breast cancer. The specific locations of these tumors were the endometrium [2], the cervix [2], melanoma [4], the esophagus [1], and the colon [1]. There were no tumors diagnosed after an ovarian cancer diagnosis. Six tumors developed after RRSO, which were located in the cervix [4] and parotid gland [1], and one melanoma was diagnosed [1].

In Table 2 the SIRs of groups of malignancies, as well as the SIRs of specific malignancies, are presented. Women in this study population were at an increased risk of developing a malignancy other than breast and ovarian cancer in general [SIR 2.25, 95%CI 1.78–2.80, *p* < 0.001]: head and neck cancer [SIR 3.17, 95%CI 1.03–7.39, *p* < 0.05], gastrointestinal cancer [SIR: 1.96, 95%CI: 1.14–3.13, *p* < 0.05], and cancer of the female genital tract, excluding ovarian and fallopian tube cancer [SIR: 2.48, 95%CI 1.61–3.65, *p* < 0.001]. For eye cancer and cancer of the central nervous system, no events were observed.

For head and neck cancer, an increased risk of tongue cancer [SIR 9.23, 95%CI 1.12–33.49, *p* < 0.05] and salivary gland cancer was found [SIR 12.2, 95%CI 1.48–44.18, *p* < 0.05]. For gastrointestinal cancer, an increased risk of stomach cancer was found [SIR 5.20, 95%CI 1.07–15.19, *p* < 0.05]. A trend for an increased risk of melanoma was observed [SIR 1.77, 95%CI 0.99–2.92, *p* < 0.05]. For cancer of the female genitals, an increased risk of cervical cancer [SIR: 3.13, 95%CI 1.56–5.60, *p* < 0.01] and uterine cancer was observed [SIR: 5.24, 95%CI 2.62–9.38, *p* < 0.001].

Twenty-four malignancies developed before the date of the DNA test of the individuals, as shown in Table 3. Of these malignancies, 10 cancers originated in the female genital tract [ovarian cancer and fallopian tube cancer excluded].

## 4. Discussion

In this cohort of 1347 women with a *BRCA1/2* GPV, 82 malignancies other than breast and ovarian cancer were detected before age 60 in 37,068 person-years at risk. Compared with women in the Dutch general population, these women have a significantly increased risk of developing malignancies in general, other than breast and ovarian cancer, before the age of 60 years. In particular, head and neck cancer, gastrointestinal cancer, and female genital cancer [other than ovarian and fallopian tube cancer] were observed more frequently than expected.

In particular, the increased risk of head and neck cancer in this population is a relatively new finding. An increased risk of pharyngeal cancer has been reported previously by Van Asperen et al. [11] for *BRCA2* GPV carriers and their untested first-degree relatives, with follow-up until the age of 80 years [RR 7.30, 95%CI 1.66–32.01]. In our cohort, the risk of pharyngeal cancer was not significantly increased [SIR 1.92, 95%CI 0.049–10.71], although the risk of tongue cancer [SIR 9.23, 95%CI 1.12–33.49] and salivary gland cancer [SIR 12.2, 95%CI 1.48–44.18] was increased. To the best of our knowledge, other studies that have included proven *BRCA1/2* GPV carriers have not reported this increased risk of head and neck cancer. Therefore, this finding warrants further exploration.

In our cohort, we also found an increased risk of gastrointestinal cancers [SIR 1.96, 95%CI 1.14–3.13], specifically stomach cancer [SIR 5.20, 95%CI 1.07–15.19]. This is consistent with the results of a meta-analysis by Lee et al. that included six studies [32], which found an increased risk of stomach cancer for *BRCA1/2* GPV carriers and/or [un]tested family members in different age groups. However, we could not confirm the increased risk of pancreatic cancer or colorectal cancer previously reported in a number of studies [16,22,24,25]. The 60-year age limit of this study may explain this difference in findings in small part, although Li et al. found an increased risk of pancreatic cancer in female *BRCA2* GPV carriers under the age of 65 years [RR = 4.92 [95%CI 2.96–7.80] for age < 65 years and 1.77 [95%CI, 0.87 to 3.58] for age ≥ 65 years]. Therefore, our small numbers may be a better explanation for the difference in findings.

The risk of developing melanoma was not significantly increased in this study, although a trend was observed [SIR 1.77, 95%CI 0.99–2.92]. An increased risk of melanoma was not found by Li et al. [25] in their large study, which included 7376 female *BRCA1* GPV carriers [RR 0.80, 95%CI 0.13–5.06] and 5032 female *BRCA2* GPV carriers [RR 1.82, 95%CI 0.43–7.71]. In the meta-analysis by Lee et al. [32], the risk of malignant melanoma was also not increased for *BRCA1* [RR 0.90, 9%CI 0.16–5.07] and *BRCA2* GPV carriers and their family members [RR 0.56, 95%CI 0.13–2.35].

However, we found an increased risk of cancers of the female genital tract, even after excluding ovarian and fallopian tube cancers [SIR 2.48, 95%CI 1.61–3.65]. Specifically, an increase in uterine cancer [SIR 5.24, 95%CI 2.62–9.38] and cervical cancer [SIR 3.13, 95%CI 1.56–5.60] was found. Previous studies have mainly focused on endometrial cancer risks in individuals with *BRCA1/2* GPV, particularly after RRSO. De Jonge et al. [19] found a two- to threefold increased risk of endometrial cancer after RRSO in *BRCA1/2* GPV carriers aged 25–80 years [SIR 2.83, 95%CI 2.18–3.65]. This risk was highest in women aged 25–40 years [SIR 9.84, 95%CI 2.68–25.2]. These results correspond with those of Segev et al. [17], who found an increased SIR of endometrial cancer in *BRCA1*, but not in *BRCA2* GPV carriers who were followed until age 75 [SIR 1.91, 95%CI 1.06–3.19 and SIR 1.75, 95%CI 0.55–4.23, respectively]. Shu et al. [20] did not find an increased endometrial cancer risk overall; however, they found a proportionally increased risk of serous/serous-like endometrial cancer after RRSO in *BRCA1* GPV carriers until age 70 [O/E 22.2, 6.05–56.9], which could not be reproduced in *BRCA2* GPV mutation carriers [O/E 6.37, 0.16–35.5]. In the meta-analysis by Lee et al. [32], the endometrial cancer risk was marginally increased for *BRCA1/2* GPV carriers and/or [un]tested family members [RR 1.53, 95% CI 1.01–2.31 for *BRCA1* and RR 1.48, 95%CI 1.24–1.78 for *BRCA2*].

For cervical cancer, Thompson et al. [16] reported an increased risk for *BRCA1* GPV carriers [RR 3.72, 95%CI 2.26–6.10]. A trend showing an increased risk of cervical cancer in *BRCA2* GPV carriers was reported by Mersch et al. [SIR 4.41, 95%CI 1.61–9.599] [24]. Lee et al. [32] did not find an increased risk for *BRCA1* or *BRCA2* GPV carriers in their meta-analysis. It has been speculated that a large portion of the uterine and cervical cancers found in *BRCA1/2* GPV carriers could be misdiagnosed ovarian cancers. However, because we made use of PALGA in the current study, as opposed to self-reporting, this is unlikely for the current study. However, detection bias could have played a role here.

Notably, *BRCA1* GPV carriers were older [median 60.8 years] than *BRCA2* GPV carriers [46.6 years] at time of linkage. The reason behind this finding is unknown. Since the identification of both breast cancer susceptibility genes was only one and a half years apart, it seems unlikely that the timing of identification is related to this age difference. However, selection bias and survival bias may have played a role in this study, since women with a diagnosis of ovarian cancer and/or breast cancer were not excluded after the date of diagnosis.

The current study is the largest single-institution study focusing on malignancies other than breast and ovarian cancer in women with a proven *BRCA1/2* GPV. Participants enrolled in our cohort all originated from the north of the Netherlands, ensuring a homogeneous group. A strength of this study is the use of data from the Netherlands Cancer Registry [NCR], which covers the entirety of the Netherlands from 1989, for the expected cancer incidence. Another strength is the use of PALGA for the observed cancers, which has excellent national coverage of histological diagnoses and provides accurate linkage of all malignancies included in this study.

The largest limitation of this study is the relatively limited number of person-years at risk available for analysis, as well as the small number of events. Risk analyses could not be performed separately for *BRCA1* and *BRCA2* GPV carriers, and the numbers are too small to draw firm conclusions on the risk estimates of specific tumors. Nonetheless, we chose to present the risk estimates of the specific tumors to compare them with those of other studies and for future [meta-] analyses. Another limitation is that information on the treatment of previous breast and tubal/ovarian cancer was unavailable and could not be corrected for in our risk analyses. Ten of the diagnosed tumors developed after a breast cancer diagnosis. Radiotherapy [33,34], chemotherapy [35], and anti-hormonal therapy [36,37] can increase the risk of developing several other types of malignancies. Tamoxifen, which is used by women with estrogen receptor-positive breast cancer, increases endometrial cancer rates with less favorable histological features and a worse survival outcomes [38]. In the current study, two uterine cancers developed after a breast cancer diagnosis, which could have increased our risk estimates for post-treatment women-years.

Furthermore, no information on lifestyle was available, although it is not to be expected that the study population has an unhealthier lifestyle than the general population. However, for some types of cancers, e.g., pharyngeal and cervical cancers, smoking status and human papillomavirus status would have been insightful to adjust for in our analyses. Lastly, it could be argued that the 24 malignancies diagnosed before DNA testing of the individual introduced testing bias, which may have influenced our results. However, none of the malignancies diagnosed before DNA testing met the referral or testing criteria outlined in the Dutch guidelines for genetic testing. Therefore, we consider the influence of testing bias in this study to be very small or not present at all.

Future research should focus on increasing the number of person-years at risk by expanding the study population. In addition, the role of previous cancer treatments and family history of cancer should be included to investigate their clinical significance and improve individualized cancer risk calculations.

## 5. Conclusions

To conclude, in this cohort of young women with a *BRCA1/2* GPV, more cases of head and neck cancer, gastrointestinal cancer, skin cancer, and female genital tract cancer were observed before the age of 60 compared to the general population. This should first be investigated in larger studies before physicians and patients are made aware of this increased risk of malignancies other than breast and ovarian cancer in female *BRCA1/2* GPV carriers.

## Figures and Tables

**Table 1:** Characteristics of the study population.

Variable	*BRCA1* GPV n = 754	*BRCA2* GPV n = 593	Total n = 1347
**Year of birth** **Median [range]**	1961 [1917–1995]	1975 [1912–1994]	1968 [1912–1997]
**Age at time of linkage ** Median [IQR]	60.8 [48.7–68.6]	46.6 [40.5–57.8]	53.9 [43.2–65.1]
**Age at DNA test ** Median [IQR]	38.9 [30.0–47.7]	41.6 [32.8–51.6]	40.3 [31.1–49.6]
**Ovarian cancer history, no. [%]**	63 [8.4%]	16 [2.7%]	79 [5.9%]
**Age at diagnosis** Median [IQR]	46.0 [33.0–55.8]	48.4 [33.9–51.6]	46.4 [33.0–55.4]
**Breast cancer history, no. [%]**	310 [41.1%]	210 [35.4%]	520 [38.6%]
**Age at diagnosis** Median [IQR]	41.2 [34.7–47.8]	46.2 [38.1–52.9]	42.7 [36.2–50.5]
History of early-age cancer other than BC and OC *, no. [%]	55 [7.5]	27 [4.6]	82 [6.1]
**Age at first diagnosis of another malignancy ** Median [IQR]	46.9 [38.5–52.2]	50.5 [44.3–53.7]	47.9 [38.9–53.1]

* Excluding BCC, basal cell carcinoma, IQR: interquartile range.

**Table 2:** Standardized incidence ratios of groups of malignancies and specific malignancies found in our study population, stratified by age and calendar time, for cancers other than breast and ovarian cancers that developed before the age of 60.

Cancer Type	Observed	Expected	Standardized Incidence Ratio [SIR]	95% Confidence Interval	*p*-Value
**All cancers** except breast and ovarian cancer	82	35.1	**2.25**	**1.78–2.80**	**<0.001**
**Head and neck**	5	1.6	**3.17**	**1.03–7.39**	**0.045**
Tongue	2	0.2	**9.23**	**1.12–33.49**	**0.04**
(Hypo)pharynx	1	0.5	1.92	0.049–10.71	0.81
Salivary glands	2	0.2	**12.2**	**1.48–44.18**	**0.02**
**Gastrointestinal**	17	8.5	**1.96**	**1.14–3.13**	**0.02**
Esophagus	2	0.5	4.43	0.54–16.00	0.15
Stomach	3	0.6	**5.20**	**1.07–15.19**	**0.04**
Colon/rectum	4	3.4	1.17	0.32–3.00	0.88
Liver	1	0.2	4.58	0.12–25.53	0.39
Pancreas	1	0.8	1.30	0.03–7.22	0.99
**Respiratory organs**	5	6.2	0.81	0.26–1.89	0.84
Lung and bronchus	5	6.1	0.83	0.27–1.93	0.87
**Skin**	16	10.6	1.50	0.86–2.44	0.15
Squamous cell carcinoma	1	1.9	0.52	0.01–2.91	0.86
Melanoma	15	8.4	1.77	0.99–2.92	0.05
**Bone, cartilage and soft tissue**	2	1.4	1.44	0.18–5.21	0.81
**Female genitals ^†^**	25	10.1	**2.48**	**1.61–3.65**	**<0.001**
Vagina, vulva	3	0.4	7.55	1.56–22.06	0.15
Cervix	11	3.5	**3.13**	**1.56–5.60**	**0.002**
Uterus	11	2.1	**5.24**	**2.62–9.38**	**<0.001**
**Urinary tract**	1	2.4	0.42	0.01–2.34	0.63
Kidney	1	1.0	0.96	0.02–5.46	0.99
**Hematological**	3	5.6	0.54	0.11–1.56	0.38
**Endocrine glands**	1	1.6	0.64	0.02–3.55	0.99
Thyroid gland	1	1.5	0.66	0.02–3.67	0.99

^†^ Excluding ovarian cancer and fallopian tube cancer. Numbers in bold represent statistically significant results.

**Table 3:** Tumors diagnosed before DNA test.

Tumor Group	Specific Tumor Location	Number of Tumors
**All cancers except breast and ovarian cancer**	NA	24
Head and neck	Tongue	1
Gastrointestinal	Liver and intrahepatic bile ducts	1
	Colon	1
	Stomach	1
	Esophagus	1
	Rectum	1
Airways	Bronchus and lung	1
Skin	Melanoma	4
Bone, cartilage and soft tissue	Connective and soft tissue	1
Female genitals ^†^	Vulva	2
	Cervix	5
	Uterine-endometrium	3
Urinary tract	NA	
Hematological	Diffuse large B-cell lymphoma	1
Endocrine glands	Parotid gland	1

^†^ Excluding ovarian cancer and fallopian tube cancer.

## Data Availability

The data generated and/or analyzed in this study are not publicly available due to privacy regulations but are available from the corresponding author upon reasonable request.
